# Supplementary material for: A Global Screen for Assembly State Changes of the Mitotic Proteome by SEC-SWATH-MS
Source: Cell Syst. 2020 Feb 26;10(2):133–155.e6. doi: 10.1016/j.cels.2020.01.001 (PMC7042714; doi:10.1016/j.cels.2020.01.001)

Q9NP58 | ABCB6_HUMAN | ABCB6 MTABC3 PRP UMAT
Monomer MW [kDa]: 93.886 Monomer expected elution fraction: 41

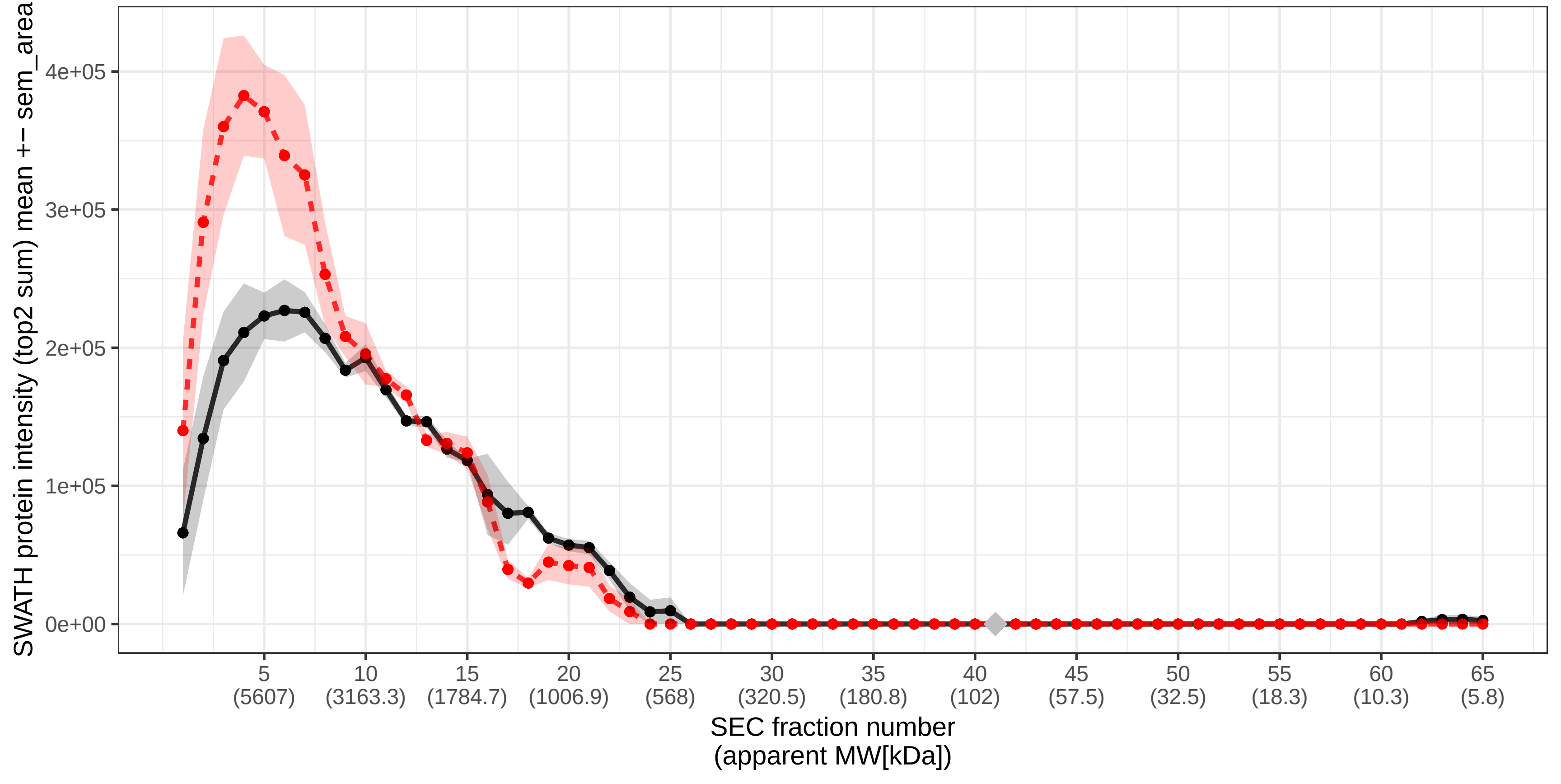

Supplement: Data S1. SEC-SWATH-MS Protein Chromatograms, Related to Figure 1 [file mmc6.zip › SECchrom_Q9NP58_ABCB6_HUMAN_ABCB6_MTABC3_PRP_UMAT.pdf]
